# Information-based methods for predicting gene function from systematic gene knock-downs

**DOI:** 10.1186/1471-2105-9-463

**Published:** 2008-10-29

**Authors:** Matthew T Weirauch, Christopher K Wong, Alexandra B Byrne, Joshua M Stuart

**Affiliations:** 1 Department of Biomolecular Engineering, 1156 High Street, Mail Stop: SOE2, University of California, Santa Cruz, CA 95064, USA; 2 Department of Molecular Genetics, The Terrence Donnelly Centre for Cellular and Biomolecular Research, 160 College St., University of Toronto, Toronto, ON, M5S 3E1, Canada

## Abstract

**Background:**

The rapid annotation of genes on a genome-wide scale is now possible for several organisms using high-throughput RNA interference assays to knock down the expression of a specific gene. To date, dozens of RNA interference phenotypes have been recorded for the nematode *Caenorhabditis elegans*. Although previous studies have demonstrated the merit of using knock-down phenotypes to predict gene function, it is unclear how the data can be used most effectively. An open question is how to optimally make use of phenotypic observations, possibly in combination with other functional genomics datasets, to identify genes that share a common role.

**Results:**

We compared several methods for detecting gene-gene functional similarity from phenotypic knock-down profiles. We found that information-based measures, which explicitly incorporate a phenotype's genomic frequency when calculating gene-gene similarity, outperform non-information-based methods. We report the presence of newly predicted modules identified from an integrated functional network containing phenotypic congruency links derived from an information-based measure. One such module is a set of genes predicted to play a role in regulating body morphology based on their multiply-supported interactions with members of the TGF-*β *signaling pathway.

**Conclusion:**

Information-based metrics significantly improve the comparison of phenotypic knock-down profiles, based upon their ability to enhance gene function prediction and identify novel functional modules.

## Background

The observable downstream effect of perturbing gene expression offers clues about a gene's normal operations in the cell. The discovery of RNA interference (RNAi) in *Caenorhabditis elegans *provides an efficient method for gene knock-down [1]. Multiple efforts have systematically knocked down large numbers of genes in *C. elegans *and scored the consequences of their disruptions using a controlled vocabulary of predefined *phenotypic descriptors *(reviewed in [2,3]). Descriptors record, for example, whether a gene knock-down causes embryonic lethality, results in uncoordinated motor control, produces altered genital structures, or gives rise to sterile worms.

Genes that participate in a common cellular function often yield similar phenotypic effects when knocked down [4-6]. The presence or absence of a set of predefined phenotypes constitutes the *phenotypic signature *of a gene, a vector of binary values that can be used to compare the observed phenotypes of one gene's knock-down to another's [7]. Large genetic networks have been constructed for both *C. elegans *[8-10] and *Saccharomyces cerevisiae *[11,12] in which genes are connected if they have highly similar (*congruent*) phenotypic signatures. Previous methods have also clustered genes based on the congruency of knock-down phenotypes [7,13]. Genes have been grouped together based on sharing a single phenotype [14,15], by visually screening for highly similar phenotypic signatures [16], by counting the number of shared phenotypes [17], by measuring the correlation of phenotypic signatures [9], and by constructing consensus phenotype profiles [18]. Some of these methods take advantage of a potential benefit of genome-wide studies: while experimenters often focus on recording the presence of specific phenotypes, the absence of phenotypes may also provide useful information for elucidating co-functional groups of genes. Although previous studies have used a variety of approaches to measure phenotypic congruency, to our knowledge a detailed evaluation of congruency metrics has not been performed. The optimal way to compare the knock-down phenotypes of genes remains an open question.

**Figure 1:**
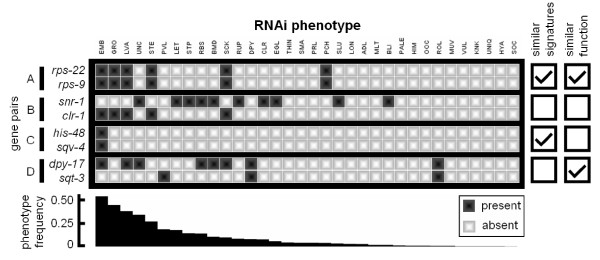
**Four possible associations between phenotypic congruency and shared gene function**. Boxes indicate that a phenotype (column) was observed to be either present (dark box) or absent (light box) upon knock-down of a gene (row) using RNA interference. Genes are grouped into four different pairs: A, B, C, and D. Pairs A and C contain genes with identical signatures; pairs A and D contain genes with related function. Phenotypes are ordered from left to right by decreasing frequency (indicated as inverted bars in the bar graph), calculated across genes with at least one phenotype.

To motivate our discussion, consider the four pairs of phenotypic signatures collected from the current *C. elegans *dataset shown in Figure 1. According to the literature, gene pairs A and D contain genes that are known to be functionally related, while B and C contain genes which are unrelated. RNAi of A's two ribosomal genes produces identical sets of phenotypic observations, which is expected for genes encoding subunits of the same protein complex. Likewise, RNAi silencing of B's unrelated genes – *snr-1*, the small nuclear RNA involved in pre-mRNA splicing and *clr-1*, a regulator of FGF signaling – produces dissimilar phenotypes. In these two cases, the similarity (or dissimilarity) of phenotypic signatures reflects the similarity (or dissimilarity) of the corresponding genes' functions.

On the other hand, there are also cases in the *C. elegans *dataset where the similarity of two phenotypic signatures may not correlate with the functions of their genes. For example, RNAi of C's unrelated genes, *his-48*, an H2B histone, and *sqv-4*, a UDP-glucose 6-dehydrogenase, has thus far resulted solely in 'Embryonic Lethality' (Emb), a common phenotype observed in roughly half of all knock-downs. In this case, although the phenotypic signatures of the genes are identical, the similarity might be purely coincidental. Conversely, the phenotypic signatures for D's two related cuticle collagen-encoding genes are considerably different. However, knock-down of both genes produces two relatively rare phenotypes, 'Dumpy' (Dpy) and 'Roller' (Rol), a combined result that is unlikely to occur by chance.

The above examples illustrate that the incorporation of phenotype background frequencies can potentially help interpret gene functional similarity from phenotypic congruency. False-positives (such as pair C) might be reduced by down-weighting common phenotypes, and true-positives (such as pair D) might be increased by up-weighting rare phenotypes.

In this paper, we systematically evaluate 19 standard metrics for measuring phenotype congruency. The metrics include well known vector-based similarity and distance measures, such as Pearson correlation and Euclidean distance. We focus on comparing *information-based *metrics, those that incorporate phenotypic frequency across the genome, to non-information-based metrics. We provide evidence that information-based metrics outperform non information-based metrics for comparing genes based on phenotype congruency. The top-scoring information-based metric rewards genes for shared *present phenotypes *(both knock-downs result in the phenotype) and *absent phenotypes *(neither knock-down results in the phenotype), while factoring in the background frequencies of both types of matches. We use the top-scoring measure to identify new functional gene modules in an integrated functional network. We conclude that information-based approaches show promise for integrating genome-wide phenotype data with other functional genomic data sources for both gene annotation and revealing new frontiers of genetic networks.

## Results

### RNA interference knock-down phenotype data

We collected data from three high-throughput RNA interference screens [19-21]. Synonymous phenotypes were merged to unite the data from these different sets (see Methods). In total, we collected data measuring the presence or absence of 34 different phenotypes under the knock-down of each of 2,376 *C. elegans *genes (Table 1). This dataset has approximate genome-wide coverage, as the majority of *C. elegans *knock-downs (about 16,000 genes) have no discernable phenotype. Thus, it is important to note that the results of comparing phenotype metrics apply only to the minority of genes that produce at least one recordable RNAi phenotype. Phenotypic signatures were recorded in a *G *× *V *binary matrix (*G *= 2,376, *V *= 34), *K*, where *K*_*gv *_was set to one if phenotype *v *was observed under the knock-down of gene *g *and was set to zero otherwise. The full matrix of compiled RNAi phenotypes is available as Additional Data File 1.

**Table 1:** The 34 phenotypes collected from high-throughput RNA interference screens.

**Full Name**	**Short Name**	**Freq.**	**Full Name**	**Short Name**	**Freq.**
Embryonic lethal	EMB	0.52	Paralyzed	PRL	0.037
Slow post-embryonic growth	GRO	0.43	Patchy appearance	PCH	0.035
Larval arrest	LVA	0.37	Sluggish appearance	SLU	0.030
Uncoordinated	UNC	0.33	Long	LON	0.026
Sterile	STE	0.26	Adult lethal	ADL	0.025
Protruding vulva	PVL	0.18	Molting defect	MLT	0.016
Lethal	LET	0.17	Blistering of cuticle	BLI	0.016
Sterile progeny	STP	0.14	Pale	PALE	0.0093
Reduced brood size	RBS	0.13	High incidence of males	HIM	0.0072
Body morphological defects	BMD	0.10	Oocytes	OOC	0.0072
Sick	SCK	0.093	Roller	ROL	0.0063
Ruptured	RUP	0.080	Multivulva	MUV	0.0059
Dumpy	DPY	0.077	Kinker	KNK	0.0013
Clear	CLR	0.074	Unique phenotype	UNIQ	0.0013
Egg-laying defect	EGL	0.056	Vulvaless	VUL	0.0013
Thin	THIN	0.040	Hyperactive	HYA	0.0008
Small	SMA	0.037	Social	SOC	0.0004

There are several sources of noise in the data, and we expect the false-negative rate to be high, mostly due to the inherent limitations of RNAi. For example, many neuronal genes are known to be refractory to RNAi. A false-negative may also occur when one phenotype excludes the observation of a second phenotype. For example, embryonic lethality could preclude observing adult phenotypes such as uncoordinated motor control. As another example, a false-positive may occur when a knock-down produces an off-target effect. To compare across congruency metrics, a complete data matrix was constructed by replacing missing values with zeros. We expect this preprocessing step to introduce a negligible amount of additional noise to the data, as the false-negative rate is likely to be much greater than the false-positive rate.

The prevalence of a phenotype, or the frequency that it is recorded for knock-downs of genes in the genome, may provide important clues for predicting gene function. Differences in phenotype prevalence may reflect technical differences (some phenotypes may be more readily detectable), biological differences (some phenotypes may result from the inhibition of several different pathways), or a combination of both. Some phenotypes are observed for hundreds of knock-downs, while others are recorded for only a few (Table 1, Additional Data File 2). Other factors, such as the mutual correlations of phenotypes with one another, might also have influence on their use in predicting gene function. As each phenotype congruency metric will incorporate (or ignore) these sources of information to varying degrees, it is not obvious which metric is optimal for comparing genes based on their phenotypic profiles.

### Evaluation of phenotype congruency metrics

A good congruency metric should assign high values to functionally-related gene pairs, while assigning low values to gene pairs which are not likely to have similar function. We calculated the phenotypic congruency between all gene pairs using 19 different metrics (Table 2), yielding a total of 2,821,500 pairwise congruency scores for each metric. We evaluated each metric based on its ability to capture associations between genes belonging to the same functional group. We used a collection of functional categories as an estimate of gene functional groups (see Methods). Category assignments created from mutant phenotype data were excluded to avoid circularity in our evaluation. We evaluated the functional coherence of each metric at the level of individual gene-gene links and at the level of gene neighborhoods.

**Table 2:** 19 metrics evaluated for their ability to identify functionally-related genes from knock-down phenotypes. See methods for mathematical definitions of each metric.

**Metric**	**Type**^a^	**Description**
*MatchPresent*	P	Counts the number of matching present phenotypes.
*MatchAbsent*	A	Counts the number of matching absent phenotypes.
*Match*	P, A	Counts the number of matching present and absent phenotypes.
*Pearson Correlation Coefficient (PCC)*	P, A	Vector correlation coefficient.
*Uncentered Pearson Correlation (UPC)*	P, A	Same as *PCC*, with vector means set to 0. Used for network construction in [9].
*UPC 2+*	P, A	Same as *UPC*, restricting to gene pairs sharing two or more phenotypes.
*Mutual Information (MI)*	P, A	Measures the degree to which knowledge about one gene's phenotypes reduces the entropy of another's.
*Euclidean Distance*	P, A	The "straight line" distance between two vectors.
*Jaccard Index*	P, A	The number of matching present phenotypes divided by the number of phenotypes present in either gene.
*Frequency Dot Product (FDP)*	P, F	Scales the number of matching present phenotypes by the frequency of each phenotype.
*Normalized FDP (nFDP)*	P, F	Same as *FDP*, but normalized by the lengths of the phenotypic signature vectors.
*Residual FDP (rFDP)*	P, F	Same as *FDP*, scaled by a score obtained by drawing random phenotypes from a Poisson distribution.
*Symmetric FDP (sFDP)*	P, A, F	Same as *FDP*, but rewards for matching absent phenotypes as well as matching present phenotypes.
*The PhenoBlast Metric*	P, A, F	Ranking system used by PhenoBlast (Gunsalus *et al*. 2004). First ranks gene pairs by *MatchPresent*, then by *MatchAbsent*, then by a metric similar to *FDP*.
*Agreement Score (AGREE)*	P, A, F	Scales the number of matching present and absent phenotypes by their frequencies across all genes [8].
*Weighted MatchPresent (wMatchPresent)*	P, C	Same as *MatchPresent*, but incorporates weights.
*Pairwise FDP (pFDP)*	P, F, C	Weights by present phenotype background pairwise co-occurences.
*Weighted FDP (wFDP)*	P, F, C	Same as *FDP*, but incorporates weights.
*Weighted AGREE (wAGREE)*	P, A, F, C	Same as *AGREE*, but incorporates weights.

^a^: The metric type indicates whether the metric rewards for shared present phenotypes (P), rewards for shared absent phenotypes (A), factors in frequencies of phenotypes across all genes (F), and/or factors in pairwise co-occurrence of phenotypes across all genes (C).

#### Functional coherence of metric links

**Figure 2:**
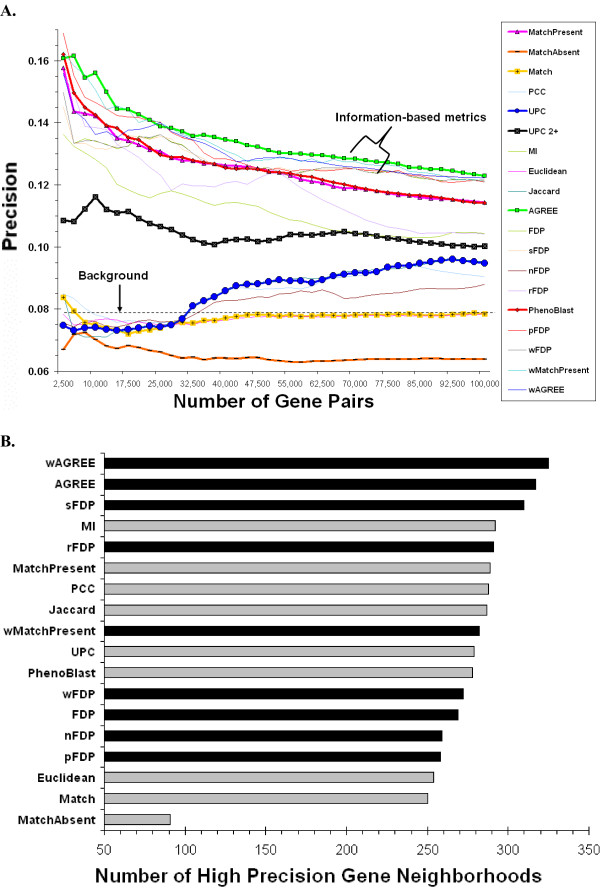
**Evaluation of metrics A. Gene network precision**. The precision of the top-scoring gene pairs is shown for each evaluated metric (see Methods). A. Gene pair precision (*y*-axis) is plotted against the number of top-scoring gene pairs for the given metric (*x*-axis). Metrics discussed in the text are displayed as bold lines. The dashed line indicates the background precision of all gene pairs in the dataset. B. Gene neighborhood functional coherence. The 25 most similar genes to each query gene were identified using each evaluated metric. The precision of these 25 gene pairs was calculated using the evaluation set. Shown is the number of query genes with high precisions (> 0.25) for information-based (black bars) and non information-based metrics (gray bars).

We first evaluated each metric by measuring how often two genes with congruent phenotypic signatures were functionally related. For a given metric, we linked two genes if their congruency score was above a threshold. We then calculated the precision of each metric for each threshold (see Methods). Overall, most metrics performed better than what would be obtained by sampling gene pairs randomly (dashed line labeled "Background" in Figure 2a). However, methods which give equal or greater weight to shared absent phenotypes (compared to shared present phenotypes) performed worse than random guessing at more stringent thresholds. For example, *MatchAbsent *(which rewards solely for shared absent phenotypes), *Match *(which rewards for shared present and absent phenotypes), *PCC *(Pearson Correlation), and *UPC *(Uncentered Pearson Correlation) all perform worse than random for networks containing 30,000 or fewer links. These results indicate that, although some information might be conveyed, shared absent phenotypes are likely to be less informative than shared present phenotypes.

Information-theoretic metrics, led by the *Agreement Score *(*AGREE*, see Methods), consistently outperformed the other metrics at most levels of network size, where network size was measured either as the number of links (Figure 2a) or as the number of genes (Additional Data File 3a). For example, when considering the top 10,000 scoring gene pairs of each metric, *AGREE *resulted in gene pairs with a precision of 0.16, which is significantly higher than previously published methods such as *The PhenoBlast Metric *(0.14, *P *< 10^-3^, proportions test) and *UPC *(0.07, *P *< 10^-5^). An evaluation of the same metrics in *Saccharomyces cerevisae *using genome-wide phenotype data downloaded from the Saccharomyces Genome Database [22] produced similar results (see Additional Data File 3b). As in worm, the information-based metrics connect yeast genes more likely to share functional relatedness compared to correlation, Euclidean distance, and counting based metrics.

The metric with the second-best performance was *The PhenoBlast Metric *[17], which first ranks genes around a query gene using the *MatchPresent *metric, then ranks ties using the *MatchAbsent *metric, and any remaining ties by an information-based metric similar to the *Frequency Dot Product *(*FDP*). It is likely that *The PhenoBlast Metric*'s ability to improve upon the *MatchPresent *metric is due to its incorporation of phenotype background frequencies into the tiebreaking step of its scoring scheme. Conversely, the *UPC *metric [9] performed poorly. As indicated by the location of the peak of the *UPC *line, the highest *UPC *precision was not obtained at the highest *UPC *value. This might be due to *UPC *linking perfectly correlated genes that share only a single frequent phenotype.

#### Functional coherence of metric neighborhoods

The functional coherence of gene pairs may be influenced by noise in the phenotype data. In order to obtain another, possibly more robust estimate of coherence than what is obtained from individual links, the functional coherence of gene neighborhoods was also assessed. Gene neighborhoods were induced for each metric by using each gene in the dataset as a query and identifying the 25 most similar genes. For each metric, we then plotted the number of gene neighborhoods with high precision (> 0.25, Figure 2b). Most metrics resulted in a similar number of high-precision neighborhoods. The *AGREE *metric resulted in the second highest number (317 neighborhoods), which is eight fewer than the similar *Weighted AGREE *metric. Similar results were obtained when using neighborhoods of different sizes, and when comparing the mean precision across query genes instead of the number of high precision genes (Additional Data File 3c). *AGREE *performed substantially better than *The PhenoBlast Metric *(278 neighborhoods), which is implemented in an online utility for the identification of gene neighborhoods with phenotypic signatures similar to a query gene [17]. Bootstrap estimates indicate that *AGREE *significantly outperformed *The PhenoBlast Metric *(*P *< 0.05, see Methods), suggesting that PhenoBlast's ranking algorithm might be improved by using the *AGREE *metric instead of its current approach.

#### Investigation of the advantages of information-based metrics

Information-based metrics reward gene pairs for sharing both present and absent phenotypes while factoring in their background frequencies. To understand why these metrics produced higher functional coherence, we identified example gene pairs where the information-based measures produced high similarity while the correlation-based approach produced low similarity, and *vice versa*.

Gene pairs illustrating each case are shown in Figure 1. Consider again the functionally-unrelated gene pair *his-48 *and *sqv-4 *(Figure 1c), which share only the frequently-occurring 'Embryonic lethal' phenotype. This pair receives a poor *AGREE *score (rank 517,524 out of 2,821,500 total gene pairs), while it receives the best possible *UPC *score. This illustrates that the incorporation of background frequencies may help to reduce false positive gene pair associations. Conversely, the phenotypic signatures of *sqt-3 *and *dpy-17*, two related collagen-associated genes, have several mismatches (Figure 1d). Nevertheless, the *AGREE *metric ranks the pair in the 99^th ^percentile (4,336 out of 2,701,650 total pairs), because the genes share two relatively rare phenotypes: 'Dumpy' (Dpy) and 'Roller' (Rol). In contrast, this gene pair is assigned a poor rank by *UPC *(831,561). This second example demonstrates that the incorporation of phenotype background frequencies can help in the identification of true-positives. Thus, the combination of reducing false-positives by down-weighting frequent phenotype coincidence and reducing false-negatives by up-weighting shared rarely occurring phenotypes may contribute to the ability of information-based methods to identify functionally similar gene pairs.

The differences in performance of the various metrics may be due to their use of properties of the phenotypes which vary in their ability to predict shared gene function. Different metrics may make either explicit or implicit use of such properties in their ability to connect functionally-related genes. To gain insights into what makes one metric better than another, we considered three properties and found that the frequency of shared function between two genes 1) increases with increasing number of shared present phenotypes; 2) increases with increasing number of shared rare present phenotypes; and 3) is unaffected by correlations between phenotypes (see Additional Data File 4).

### Network topology comparison

Based on its high performance relative to the other information-based metrics, we chose the *AGREE *metric to help provide novel insights into the systems biology of *C. elegans*. We compared a phenotype congruency network constructed using the *AGREE *metric (ANET) to a network constructed with a previously published method. In a recent study, Gunsalus and colleagues demonstrated the utility of combining phenotype congruency with other high-throughput data for the identification of 'molecular machines' [9]. The authors constructed a *UPC*-induced phenotype congruency network (UNET) by linking gene pairs with *UPC *values of 0.50 or higher. Due to our larger dataset, a *UPC *threshold of 0.50 results in a network in which a quarter of all possible gene pairs are connected, which is not specific enough for practical use. Therefore, we constructed the UNET by linking genes with *UPC *scores exceeding 0.866, which corresponds to a *UPC *significance of *P *< 0.01 (see Methods). We chose an *AGREE *threshold such that the same number of gene pairs (32,530) was included in the ANET. The resulting networks have precisions of 0.13 (ANET) and 0.07 (UNET).

We first compared general topological features of the networks. We found that the ANET and UNET exhibit different connectivity. On average, genes in the ANET are linked to 53 other genes (+/- 47), while genes in the UNET are linked to 34 other genes (+/- 49). The ANET is comprised of one large connected component containing 1,202 genes, and six smaller connected components containing the remaining seventeen genes. In contrast, the UNET is comprised of a large connected component, containing 601 genes (25% of the original gene set), and 159 smaller components, all but eight of which are fully connected. 17 of the fully-connected UNET components (11%) consist of gene pairs with identical phenotypic signatures that contain only a single knock-down phenotype, which results in a perfect *UPC *score. For example, the largest connected components include a component of 173 genes whose knock-downs result solely in an 'Embryonic lethal' (Emb) phenotype, a component of 99 genes with only a 'Slow post-embryonic growth' (Gro) phenotype, and a component of 50 genes with only an 'Uncoordinated' (Unc) phenotype. These gene pairs which share only a single frequently-occurring phenotype are unlikely to be co-functional, as evidenced by the precisions of these connected components (0.07, 0.04, and 0.08, respectively).

**Figure 3:**
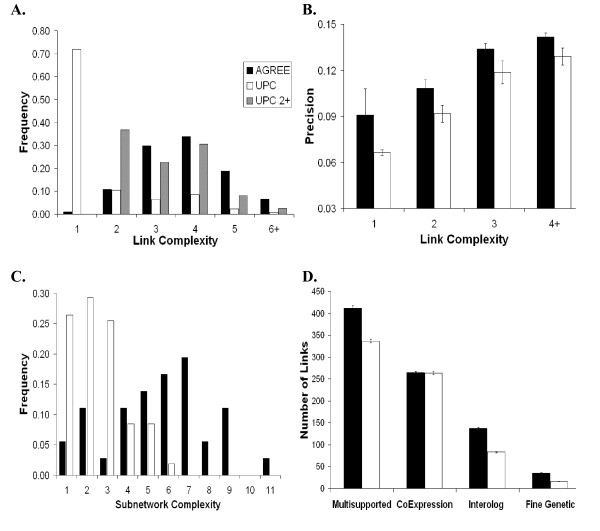
**Network and subnetwork comparisons**. Key is shown in the upper left-hand panel. A. *AGREE *links join genes with more shared present phenotypes. The *x*-axis indicates the *link complexity*, or number of shared present phenotypes. The *y*-axis indicates the frequency of occurrence of each link complexity bin. B. *AGREE *links have higher precision for every level of link complexity. The links of each network were binned by their complexity (*x*-axis). The *y*-axis indicates the precision of each bin for each network. Error bars indicate one standard deviation, assuming a binomial distribution. C. *AGREE *subnetworks are enriched for more phenotypes. The number of over-represented phenotypes present in the genes of each subnetwork (*subnetwork complexity*) was determined using the hypergeometric distribution (see Methods). The *x*-axis indicates subnetwork complexity. The *y*-axis indicates the frequency of the given subnetwork complexity in the ANET and UNET. D. A greater number of *AGREE *links are supported by other data types. The *x*-axis indicates the data type. The *y*-axis indicates the number of links in the ANET and UNET which are supported by that data type. Error bars indicate one standard deviation, assuming a binomial distribution.

We next sought to compare the number of shared present phenotypes between genes linked in the ANET and UNET. To quantify this, we define the *complexity *of a link to be the total number of present phenotypes co-induced by a pair of genes. We refer to a link with a complexity of two or more as a *complex link*, and otherwise a *simple link*. We found that the ANET connects genes exhibiting a higher number of phenotypes than genes connected in the UNET (Figure 3a). Most of the links in the ANET are complex (median complexity of four), while most links in the UNET are simple (median complexity of one). The ANET contains a total of 330 simple links (1% of the network), where each simple link connects genes sharing one rare phenotype (phenotype frequency < 4%). Conversely, there are 23,375 simple links (72% of the network) in the UNET. Almost two-thirds (14,878) of these links connect genes sharing the 'Embryonic lethal' phenotype (Emb). We also measured the complexity of the UNET after removing simple links from consideration. The resulting UNET still contained links with lower complexity than the ANET (black bars, Figure 3a). Additionally, for each level of complexity, the ANET contains gene pairs with higher precision than the UNET (Figure 3b). These results indicate that *AGREE *not only identifies links of higher complexity, but also links of greater biological relevance.

The networks induced by *AGREE *and *UPC *are orthogonal, having little overlap in their gene pairs (only 1,909 links, or 5.8% of the networks). These shared links have a precision of 0.158. The links unique to the ANET have a precision of 0.134, whereas links unique to the UNET have a precision of 0.075, which is significantly less than the precision of the shared links (*P *< 10^-38^, proportions test). Taken together, these results indicate that the *AGREE *metric not only identifies many gene associations that are missed by the *UPC *metric, but also finds associations that are of an overall higher quality.

**Figure 4:**
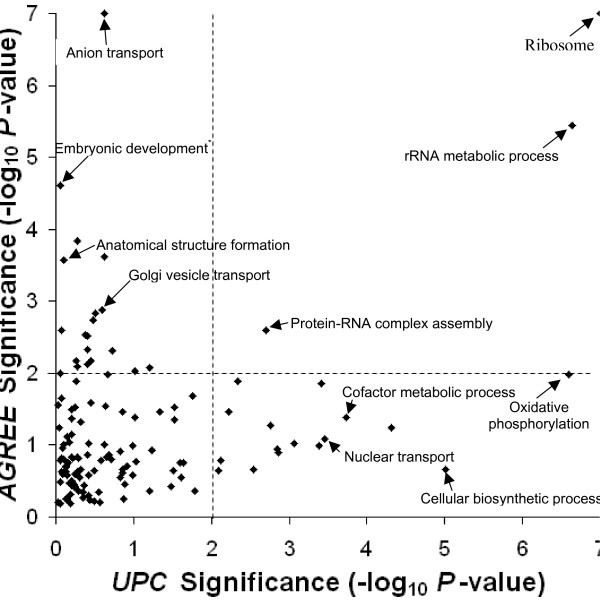
**Comparison of *AGREE *and *UPC *scores within functional categories**. Each point represents one functional category, and indicates the negative log significance of the pairwise scores of all genes within that functional category using the *AGREE *(*x*-axis) and *UPC *metrics (*y*-axis). Dashed lines indicate significances of *P *< 0.01 or better. Categories were taken from the evaluation set, and were filtered to ensure that no two categories overlap by greater than half of their gene members (see Methods). * 'Embryonic development' is short for 'Embryonic development ending in birth or egg hatching.'

While the set of gene pairs in the ANET and UNET are different, it was still possible that the metrics brought genes into proximity from the same pathways, albeit through different gene pairs. However, we found that pathways containing high-scoring *AGREE *gene pairs are largely not the same as the pathways containing high-scoring *UPC *gene pairs. We created a non-redundant set of functional categories from our evaluation set, and calculated the significance of the pairwise scores of its gene members for each category using the *AGREE *and *UPC *metrics (see Methods). This calculation assigns high scores to categories whose gene members have significantly similar phenotypic signatures according to the metric. Figure 4 plots the highest significance achieved for each non-redundant category using each metric. Overall, three housekeeping-related categories were enriched for high scores using both metrics:'Ribosome', 'rRNA metabolic process', and 'protein-RNA complex assembly.' 20 classes were exclusively enriched for *AGREE*, and 16 were enriched only for *UPC *(Additional Data File 5). Overall, the *AGREE *metric captured several categories involved in development and cell structure, while the *UPC *metric identified categories involved in translation and other protein expression-related activities. Thus, the genes that are brought into proximity by the two metrics largely belong to complementary biological processes.

### Comparison of subnetworks

Decomposing large interaction networks into their constituent subnetworks has proven useful for the elucidation of genetic pathways [23]. As the majority of links in the ANET and UNET connect different pairs of genes, we investigated how the different link content of the networks could influence subnetwork identification, which in turn could influence the prediction of functional modules. To detect subnetworks, we used the MODES algorithm [24] (see Methods), and identified a total of 37 ANET subnetworks and 107 UNET subnetworks. ANET subnetworks contained 33 genes on average (+/- 20), while UNET subnetworks were smaller, containing 14 (+/- 21) genes. For each UNET subnetwork, we determined if its genes had a significant number of links in the ANET. The density of the links within a subnetwork was assessed using a connectivity score calculated as the fraction of links connecting the genes of the subnetwork out of the total possible number of such links. The significance of the score was estimated for each subnetwork size using a random sampling procedure (see Methods).

As expected, the ANET and UNET share many small (7 +/- 6 genes), low-complexity (2.6 +/- 1.3) subnetworks in which the genes share one or two rare phenotypes. Even though the number of shared subnetworks amounts to half (51 out of 107) of the subnetworks identified in the UNET, the total fraction of network links included in these subnetworks is small (approximately 5%). The subnetworks unique to the UNET are much larger (21 +/- 27 genes) and lower in complexity (2.1 +/- 1.1), whereas the majority of ANET subnetworks are enriched for five or more phenotypes (Figure 3c). The overall difference in complexities is significant based on bootstrap resampling (*P *< 10^-12^, see Methods). Thus, both the ANET and UNET produce a similar number of small subnetworks of genes sharing a single rare phenotype. However, the majority of genes and links in the UNET reside in large subnetworks that are not well-connected in the ANET.

### Integration into a *C. elegans *genetic network

Previous studies have demonstrated the utility of combining phenotype congruency data with other data types in order to identify functional relationships [8,9,13,25]. We investigated how the ANET integrates with other data types, including interactions based on coexpression, protein-protein interactions, and a compendium of low-throughput genetic interactions (see Methods). To this end, we took the union of all of these interactions with the ANET links to create a superimposed *AGREE *network (available as Additional Data File 6). In an identical manner, a superimposed *UPC *network was constructed using UNET links.

We first investigated *AGREE*'s ability to identify gene associations supported by other data sources and found that significantly more ANET links were *multiply-supported *links (present in multiple data sources) compared to UNET links (23% increase, see Figure 3d and Additional Data File 6). Furthermore, the ANET performs comparably to, or better than, the UNET for each data type considered separately (Figure 3d). In a manner similar to the approach by Gunsalus *et al*. [9], we restricted our analysis to multiply-supported links. This revealed that only 81 of 412 (20%) multiply-supported ANET links were also supported by UNET links. The 412 multiply-supported ANET links have a precision of 0.607, while the 336 multiply-supported UNET links have a precision of 0.485. Thus, the multiply-supported ANET contains gene interactions that are different from, but of higher quality than, the multiply-supported UNET.

We next searched for gene modules composed of multiple data types that are undetectable using the *UPC *metric. We first identified multiply-supported subnetworks (MSSNs) using previously published methods [8] (see Methods). A total of 29 *AGREE *and 50 *UPC *MSSNs were identified with phenotype congruency links as one of the enriched link types (provided in Additional Data File 7). Many of these MSSNs represent functionally coherent gene groups, as 27 (93.1%) *AGREE *MSSNs and 47 (94.0%) *UPC *MSSNs are enriched for at least one category in our validation set (see Methods). 14 of the *AGREE *MSSNs represent novel gene combinations with respect to the *UPC *metric (see Methods and Additional Data File 7). All of these unique subnetworks are enriched for at least one functional category, indicative of their potential in the discovery of novel gene modules.

**Figure 5:**
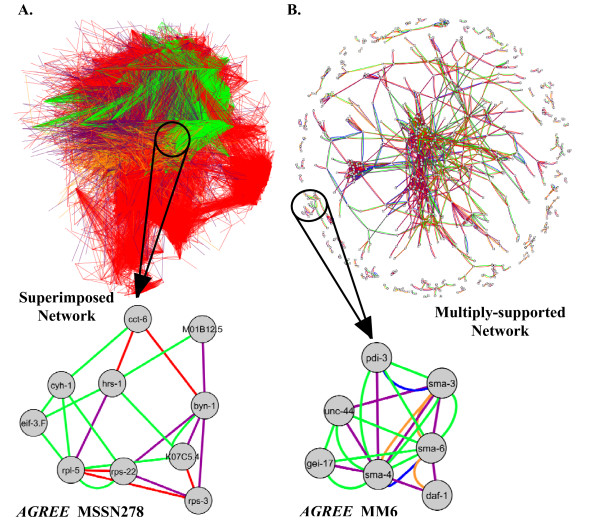
**Integration with other data sources**. Link colors indicate interaction type: Green, *AGREE *phenotype congruency; Blue, *UPC *phenotype congruency; Purple, protein-protein; Red, co-expression; Orange, genetic. A. Superimposed network. Superimposed network created from multiple data sources. Shown below is a multiply-supported subnetwork identified in the superimposed network. B. Multiply-supported network. Network created from restricting to links supported by multiple data sources. Shown below the network is one 'molecular machine', identified as a subnetwork in the multiply-supported network.

Module MSSN278 (Figure 5a) contains ten genes, seven of which are annotated as being involved in ribosomal biogenesis. Many of the genes in this module are components of the core translational machinery. Other genes in the module are predicted to be necessary for ribosome synthesis, including *byn-1 *[26], K07C5.4 [27], and *eif-3.F *[27]. Furthermore, two of the three genes that are not annotated with ribosomal function or biogenesis are predicted to be involved in translation. *hrs-1 *is a predicted histidyl-tRNA synthetase, and *cct-6 *(Chaperonin Containing TCP-1) is a cytosolic chaperonin involved in folding of nascent proteins [27,28]. Since many developmental pathways are regulated by translation, it is not surprising that disruption of the genes in this module results in multiple severe phenotypes such as 'Sterile', 'Embryonic lethal', 'Larval arrest', 'Slow growth', and 'Sick'. Thus, the *AGREE *metric correctly identifies components of the translational machinery which the *UPC *metric is unable to identify, due to the multiple phenotypes of the genes in this module being present in slightly differing combinations.

We also identified modules of densely connected genes in the multiply-supported superimposed network using a module discovery method similar to the method used by Gunsalus and colleagues to detect 'molecular machines' [9]. We identified a total of 112 molecular machines, and labeled them with the prefix 'MM' (Additional Data File 7). Module MM6 (Figure 5b) contains seven body morphology-related genes with similar phenotypic signatures supported by multiple functional sources. Three of the genes in the module, *sma-3 *(Smad) [29], *sma-6 *(type I TGF-*β *receptor) [30], and *sma-4 *(Smad) [29] are TGF-*β *pathway components that play a role in body size and male tail development. Both *gei-17*(GEX Interacting protein) and *pdi-3 *(Protein Disulfide Isomerase), are thought to regulate tissue morphology [31]. The presence of the 'Small' phenotype and, importantly, the lack of other common phenotypes, enables *AGREE *to connect genes that regulate different aspects of morphology which are missed by *UPC*. *UPC *fails to connect most of the *sma *genes because some of them are also annotated with the 'Dumpy' phenotype, and others are not.

Finally, we estimated the predictive influence of adding phenotype congruency links to the integrated *C. elegans *network of Lee *et al*. (2008) [32]. We found that the log-likelihood scores (LLS) placed significantly more weight on the AGREE-derived links, as compared to the UPC-derived links (see Additional Data File 8 for the regression of each metric onto the LLS score). 388 AGREE links were above the LLS cutoff of 1.5, and would therefore be included into the Lee *et al*. network. In contrast, none of the UPC links have sufficiently high LLS scores on their own to merit inclusion. Nearly all of the AGREE links (384 out of 388) represent new connections into the Lee *et al*. integrated network. Thus, the phenotypes provide a small, but appreciable set of new connections not elucidated by the combination of the other data sources.

## Discussion

We compared the ability of several metrics to connect functionally related genes using high-throughput RNAi knock-down phenotype data. We found that the *Agreement Score *(*AGREE*) performed better than 18 other metrics in this regard. Additionally, with respect to the previously published *UPC *metric, the phenotype congruency network induced by the *AGREE *metric connects genes with more shared phenotypes, and contains more links that are supported by additional data sources.

While the *AGREE *metric outperforms the metrics tested in this study, the results provide information about how it could be improved upon. For example, although the frequency of a phenotype does carry some information, placing too much weight on rare phenotypes may lead to erroneous functional predictions. This may explain why *AGREE *outperformed the similar information-based metric *Symmetric Frequency Dot Product *(*sFDP*). To illustrate, let *IDF*_*v *_be the inverse document frequency of phenotype *v*. The component of the *AGREE *metric that rewards for shared present phenotypes is a sum over the set {*IDF*_*v*_}, while *sFDP *is a sum over {*IDF*_*v*_^2^}. Our results indicate that squaring the inverse frequencies may over-emphasize rare phenotypes. Thus, the exponent in the summation could be parameterized to give the generalized sum over the set {*IDF*_*v*_^*p*^}. For example, using this notation, *Match *corresponds to *p *= 0. The observation that *Match *outperformed *sFDP *indicates that one might find an improvement over the *AGREE *metric by optimizing *p *between *0 *and *1*. Likewise, a corresponding parameter could be optimized for the component of *AGREE *which rewards for shared absent phenotypes.

Recently, approaches inspired by natural language processing (NLP) have been applied to a range of biological problems (reviewed in [33]). In this study, the task of finding related genes based on common phenotypic consequences can be likened to the task of identifying similar documents based on the presence and absence of particular words. Other studies have proposed NLP-based methods for protein interaction prediction (reviewed in [34]), protein subcellular localization prediction [35], prediction of gene function (reviewed in [36]), GO-based protein semantic similarity calculation [37], and the identification of candidate genes for complex traits [38].

For this study, we focused on the analysis of high-throughput RNAi phenotype data in *C. elegans*. Such data is currently also being generated for other species, most notably in *S. cerevisiae *(e.g. the morphological traits examined in [39]) and *D. melanogaster *(reviewed in [40]). Our results on *S. cerevisiae *(see Additional Data File 3b) suggest that metrics such as the ones evaluated in this paper may be useful for analyzing data from other species.

Finally, all of the metrics evaluated in this study are limited in the sense that they treat phenotypes as vectors, ignoring any known or latent relationships that may exist among the phenotypes. Rather than use binary presence/absence calls, comparisons could make use of quantitative values representing "strengths" if replicates of gene-phenotype observations are available either within or across studies. Additionally, the introduction of more formal knowledge representations for *C. elegans *that describe *is-a *or *part-of *relations among phenotypes, such as the Mammalian Phenotype Ontology [41], may allow comparisons that incorporate semantic structure such as the work by Pesquita *et al*. (2008) [37].

## Conclusion

With the maturation of RNAi technology, it is now possible to knock down genes in a high-throughput manner. We found that an information-based measure, which we call the *AGREE *metric, outperforms other tested metrics and that its use in the construction of a superimposed network containing data from several sources results in high quality predictions, many of which go undetected using other methods. Other measures of phenotype congruency that we did not test may outperform those tested in this study. Our results indicate that metrics borrowed from the field of natural language processing may prove useful in this domain. We suggest that metrics which incorporate the frequency of shared phenotypes, such as the *AGREE *metric, should be used in place of unweighted correlations for functional genomics analyses involving RNAi knock-down data.

## Methods

### RNA Interference-Induced Phenotype Data

An RNA Interference (RNAi)-induced phenotype compendium was assembled by compiling the results of three genome-wide RNAi studies: 30 phenotypes scored for 1,470 genes from [19]; 27 phenotypes scored for 1,778 genes from [21]; and 26 phenotypes scored for 1,066 genes from [20]. Several phenotype annotations in the datasets were converted to provide a uniform language which allowed the three datasets to be integrated. These conversions included labeling brood counts scored as "1–5" and "6–10" as "Ste"; re-labeling "Ppz" as "Prl"; re-labeling "Lvl" as "Let"; labeling any embryonic lethal percentages over 10% as "Emb"; and re-labeling "Slm" and "Thn" as "Thin". In total, 34 phenotypes scored across 2,376 unique genes were collected from the three studies and recorded in a 2,376-by-34 RNAi phenotype matrix, *K*. Each entry in the matrix, *K*_*iv*_, was set to 1 if RNAi against gene *i *produced phenotype *v *in at least one of the three studies and was set to 0 otherwise. We refer to each row in the matrix, *K*_*i*_, as a *phenotypic signature*.

### Calculation of congruency metrics

We calculated the similarity of phenotypic signatures using a variety of congruency metrics. For binary vectors such as those used in this study, several metrics are equivalent, including (*Euclidean Distance *and *Canberra Distance*) and (*Match*, *Hamming Distance*, and *Rand Index*). In such cases, the results of only one metric from each group are presented. Additionally, several metrics are conceptually and mathematically similar. In such cases, the metric with the highest precision curve (as shown in Figure 2a) was chosen for presentation. To simplify discussion, we group the metrics into four categories: match-based metrics, unweighted metrics, metrics weighted by phenotype frequencies, and metrics incorporating phenotype correlations.

To facilitate direct comparison, each pairwise similarity between genes *i *and *j*, *s*(*i*, *j*) was re-scaled to reside between zero and one:

$$Re\text{-}scaled(s(i,j))=\frac{s(i,j)-\min\left(s(i,j)\right)}{\max\left(s(i,j)\right)-\min\left(s(i,j)\right)},$$

where min(*s*(*i*, *j*)) and max(*s*(*i*, *j*)) are the minimum and maximum unnormalized score computed across all gene pairs, respectively. All metrics tested were similarity measures, in which a higher score for a gene pair indicated a higher degree of relatedness.

#### Match-based metrics

One way to compare two phenotypic signatures is to count the number of matching phenotypes. We evaluated three such match-based metrics.

*MatchPresent *counts the number of matching present phenotypes between a gene pair:

$$MatchPresent(i,j)=\sum_{v=1}^{34}K_{iv}K_{jv}.$$

*MatchAbsent *counts the number of matching absent phenotypes between a gene pair:

$$MatchAbsent(i,j)=\sum_{v=1}^{34}\left(1-K_{iv})(1-K_{jv}\right)$$

*Match *counts the number of matching present and absent phenotypes between a gene pair:

$$Match(i,j)=\sum_{v=1}^{34}K_{iv}K_{jv}+(1-K_{iv})(1-K_{jv}).$$

#### Unweighted metrics

We categorize metrics as unweighted if they operate on the input binary vectors without incorporating additional statistics on phenotype background frequencies or phenotype correlations. The following five unweighted metrics were tested.

### Pearson Correlation Coefficient (PCC)

The *Pearson Correlation Coefficient *(*PCC*) measures the departure of two variables from independence. In our notation, the PCC definition is:

$$PCC(i,j)=\frac{\sum_{v=1}^{34}\left(K_{iv}-{\bar{K}}_{i}\right)\left(K_{jv}-{\bar{K}}_{j}\right)}{\sqrt{\sum_{v=1}^{34}{\left(K_{iv}-{\bar{K}}_{i}\right)}^{2}\sum_{v=1}^{34}{\left(K_{jv}-{\bar{K}}_{j}\right)}^{2}}},$$

where ${\bar{K}}_{i}$ is the mean across the phenotypic signature of gene *i*. A positive value for a gene pair (*i*, *j*) indicates that the phenotypes present in gene *i *also tend to be present in gene *j*, and phenotypes absent in gene *i *also tend to be absent in gene *j*.

### Uncentered Pearson Correlation (UPC)

The *Uncentered Pearson Correlation *(*UPC*) was used as a phenotype congruency metric for network construction in (Gunsalus *et al*. 2005). The *UPC *is the same as *PCC*, but does not center the vectors around their means. In our notation, the UPC definition is:

$$UPC(i,j)=\frac{\sum_{v=1}^{34}K_{iv}K_{jv}}{\sqrt{\sum_{v=1}^{34}{\left(K_{iv}-{\bar{K}}_{i}\right)}^{2}\sum_{v=1}^{34}{\left(K_{jv}-{\bar{K}}_{j}\right)}^{2}}}$$

### Mutual Information

*Mutual Information *(*MI*) is an information theoretic quantity that expresses the mutual dependence between two random variables. It measures the degree to which knowledge about one random variable reduces the entropy of another random variable. In our notation, the mutual information is:

$$MI\left(i,j\right)=\frac{1}{34}\sum_{a=0}^{1}\sum_{b=0}^{1}N_{ijab}\log_{2}\left(\frac{34N_{ijab}}{N_{ia}N_{jb}}\right),$$

where *N*_*ijab *_is the number of times genes *i *and *j *took on values *a *and *b *together, and *N*_*ia *_is the number of times gene *i *took on value *a*. For example, *N*_11 _is the number of present phenotypes of gene 1, and *N*_1200 _is the number of times gene 1 and gene 2 together had absent phenotypes.

### Euclidean Distance

In our notation, the *Euclidean Distance *(*Euclidean*) between two phenotypic signatures in *V*-dimensional space, where *V *= 34, is:

$$Euclidean(i,j)=\sqrt{\sum_{v=1}^{34}{\left(K_{iv}-K_{jv}\right)}^{2}}.$$

We converted this metric to a similarity metric by using 1 – *Re-scaled*(*Euclidean*(*i*, *j*)).

### Jaccard Index

The *Jaccard Index *(*Jaccard*) is commonly used in clustering applications. It compares the number of shared present phenotypes to the number of phenotypes present in either gene:

$$Jaccard(i,j)=\frac{\sum_{v=1}^{34}K_{iv}K_{jv}}{\sum_{v=1}^{34}\left(K_{iv}+K_{jv}-K_{iv}K_{jv}\right)}.$$

#### Information-based metrics

**Figure 6:**
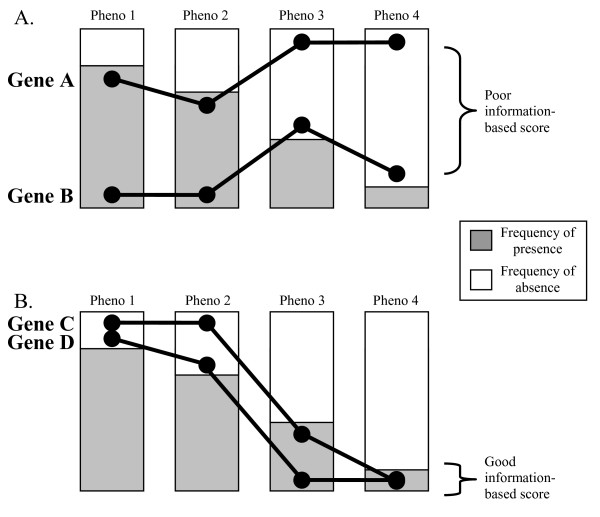
**Illustration of frequency-weighted phenotype congruency**. The distance between points reflects the relative number of genes that share (or lack) the corresponding phenotype. Phenotypic signatures for a single gene are represented as a line; phenotypes correspond to individual vertical bars. The length of the gray area in a phenotype's bar is proportional to its frequency. The presence of a phenotype for a gene is indicated by drawing a point at the extremes of the shaded area for the phenotype. The total distance between the lines reflects the relative dissimilarity of the gene pair. A. Two genes which share frequently occurring present and absent phenotypes, and so would receive a poor information-based score. B. Two genes which share rare present and absent phenotypes, and so would receive a good information-based score.

In this paper, information-based metrics are defined to be metrics that factor in the background frequency of matching present and absent phenotypes. To illustrate their utility, consider the scenario of scoring two pairs of genes: (A, B) and (B, C), where each pair of genes shares two present phenotypes and two absent phenotypes. Using a non-information based metric, each pair of genes will receive an equivalent score, as each has an equal number of matches. Using an information-based metric, (A, B) will receive a poor score, as this pair shares present and absent phenotypes which occur more frequently (Figure 6a). Conversely, (C, D) will receive a high information-theoretic score, as the genes in the pair share less frequent present and absent phenotypes (Figure 6b).

For the following definitions, let *f*_*v *_denote the frequency of phenotype *v *across all genes in the knock-down matrix *K*.

#### Agreement Score (AGREE)

The *Agreement Score *(*AGREE) *measures the bits of information that are encoded in the phenotypes shared by two genes, and is defined as:

$$AGREE\left(i,j\right)=\sum_{v=1}^{34}S_{p}(i,j,v)+S_{a}(i,j,v),$$

where

$$S_{p}(i,j,v)=K_{iv}K_{jv}\log_{2}\left(\frac{1}{f_{v}}\right)$$

and

$$S_{a}(i,j,v)=\left(1-K_{iv}\right)\left(1-K_{jv}\right)\log_{2}\left(\frac{1}{1-f_{v}}\right).$$

*S*_*p *_and *S*_*a *_reward a gene pair for shared present and absent phenotypes, respectively. *AGREE *was used in the construction of a *C. elegans *phenotype network, and called *Loss-of-Function Agreement Score *in[8]. If RNAi produces phenotype *v *in two genes, the *AGREE *score will increase by $\log_{2}\left(\frac{1}{f_{v}}\right)$ bits. The $\log_{2}\left(\frac{1}{f_{v}}\right)$ term is known as the *Inverse Document Frequency (IDF)*, which is often used in natural language processing applications [42].

#### Frequency Dot Product (FDP)

The dot product is frequently used to measure the similarity between two IDF vectors [42], and is here referred to as the *Frequency Dot Product *(*FDP*). *FDP *weights each present phenotype shared between two genes by the frequency of occurrence of that phenotype across all genes. We define the *FDP *as:

$$FDP\left(i,j\right)=\sum_{v=1}^{34}S_{p}{\left(i,j,v\right)}^{2},$$

where *S*_*p *_is defined as above for *AGREE*.

#### Symmetric FDP

We also define the *Symmetric FDP *(*sFDP) *to reward shared absent phenotypes in the same way as shared present phenotypes. In our notation this measure is defined to be:

$$sFDP\left(i,j\right)=\sum_{v=1}^{34}S_{p}{\left(i,j,v\right)}^{2}+S_{a}{\left(i,j,v\right)}^{2},$$

where *S*_*p *_and *S*_*a *_are defined as above for *AGREE*.

#### Normalized FDP

The *Normalized FDP *(*nFDP*) is equivalent to *FDP*, except that the measure is normalized by the lengths of the two vectors:

$$nFDP(i,j)=\frac{FDP(i,j)}{\left|K_{i}\right|\times \left|K_{j}\right|}.$$

*nFDP *is the cosine similarity measure computed on IDF-weighted vectors. Normalizing the dot product by the lengths of the vectors penalizes gene pairs which cause many knock-down phenotypes.

#### Residual FDP

The *Residual FDP *(*rFDP*) is frequently used for the identification of relevant terms in Natural Language Processing [42]. The rFDP is defined to be the difference between the *FDP *score of a gene pair and its expected *FDP *score as given by a Poisson distribution. In our notation, the *rFDP *between two phenotypic signatures is:

$$rFDP\left(i,j\right)=\sum_{v=1}^{34}{\left(S_{p}(i,j,v)-S_{penalty}(v)\right)}^{2},$$

where *S*_*p *_is defined as above for *AGREE*, and

$$S_{penalty}(v)=K_{iv}K_{jv}\log_{2}\left(\frac{1}{1-e^{-f_{v}}}\right).$$

#### The PhenoBlast Metric

*The PhenoBlast Metric *is used to rank genes based on the similarity of their phenotypic signatures to the profile of a query gene. This method first ranks genes by the *MatchPresent *metric, then by the *MatchAbsent *metric, and finally by a metric similar to the *FDP *metric, as defined in [17].

### Metrics factoring in phenotype correlations

#### Pairwise FDP

The *Pairwise FDP *(*pFDP*) rewards gene pairs that share rarely co-occuring present phenotypes. *pFDP *thus incorporates a correction based on phenotype correlation. For example, it gives a higher score to genes sharing rarely co-occuring pairs of phenotypes like 'Long' and 'Ruptured' compared to a pair sharing commonly co-ocurring phenotypes like 'Embryonic lethal' and 'Lethal' phenotypes. We define *pFDP *as:

$$pFDP(i,j)=\sum_{u=1}^{34}\sum_{v=u+1}^{34}{\left[S_{co-p}(i,j,u,v)-\lambda b(i,j)\right]}^{2},$$

where

$$S_{co-p}(i,j,u,v)=K_{iu}K_{ju}K_{iv}K_{jv}\log_{2}\left(\frac{1}{f_{uv}}\right)$$

and

$$b(i,j)=\sum_{v=1}^{34}S_{p}(i,j,v).$$

*S*_*p *_is defined as above for *AGREE*, and *f*_*uv *_is the background co-occurrence of phenotypes *u *and *v*. The second term in the *pFDP *equation corrects phenotype pairings by the background frequency of each present phenotype. We used a value for *β *equivalent to half the number of shared present phenotypes between genes *i *and *j*.

#### Weighted FDP

The *Weighted FDP (wFDP) *is similar to *FDP*, but each matching present phenotype is inversely weighted to its co-occurrence with other matching present phenotypes across all genes. We calculated the *wFDP *of a gene pair as

$$wFDP\left(i,j\right)=\sum_{v=1}^{34}w_{ijv}S_{p}{\left(i,j,v\right)}^{2},$$

where *S*_*p *_is defined as above for *AGREE*.

The weights *w*_*ijv *_are pre-computed across all genes as:

$$w_{ijv}=\sum_{w=1}^{34}K_{iv}K_{jv}K_{iw}K_{jw}\frac{1}{f_{vw}},$$

where *f*_*vw *_is the background co-occurrence of phenotypes *v *and *w*.

#### Weighted MatchPresent

The *Weighted MatchPresent *metric (*wMatchPresent*) counts the number of matching present phenotypes between a gene pair, weighting each shared present phenotype by the degree to which it does not co-occur with other shared present phenotypes across all genes:

$$wMatchPresent(i,j)=\sum_{v=1}^{34}w_{ijv}K_{iv}K_{jv}.$$

The weights *w*_*ijv *_are pre-computed across all genes as described above for *Weighted FDP*.

#### Weighted AGREE

The *Weighted AGREE *metric (*wAGREE*) weights each phenotype by the degree to which it does not co-occur with other matching phenotypes across all genes:

$$wAGREE\left(i,j\right)=\sum_{v=1}^{34}w_{ijv}S_{p}(i,j,v)+z_{ijv}S_{a}(i,j,v),$$

where *S*_*p *_and *S*_*a *_are defined as above for *AGREE*.

The weights *w*_*ijv *_are pre-computed as described above for *Weighted FDP*. The weights *z*_*ijv *_are calculated in the same way as the weights *w*_*ijv*_, but for matching absent phenotypes instead of matching present phenotypes (i.e. counting matching zeroes in each phenotypic signature instead of ones).

### Evaluation set

We evaluated each congruency metric by assessing its ability to link gene pairs known to share a similar function. We created positive and negative evaluation sets from biological process annotation databases. The positive set consisted of the union of the set of all gene pairs that share a common category in any of the three following sets: 1) *C. elegans *Gene Ontology (GO) [43] process categories containing 200 or fewer genes, restricting to gene associations not created from *C. elegans *knock-down phenotype data (i.e. removing all gene/category associations with the "inferred from mutant phenotype" (IMP) evidence code); 2) GO process categories of size 200 or smaller mapped from *H. sapiens*, *M. musculus*, *D. melanogaster*, and *S. cerevisiae*. Orthology mappings were created from reciprocal-best BlastP [44] hits; or 3) genes in *C. elegans *metabolic pathways, as reported in the Kyoto Encyclopedia of Genes and Genomes [45]. Because more than 26,000 *C. elegans *GO associations based on mutant phenotypes were removed to reduce circularity in the evaluations, we included GO annotations predicted from other species in order to increase the size of the effective positive set. While the inclusion of GO annotations from other species is likely to result in an increase in false positives, we do not expect them to be introduced in such a manner that would bias one metric in favor of another.

The negative evaluation set consisted of the union of the sets of all gene pairs contained in any GO process, Orthologous GO process, or KEGG category, removing any pairs that are members of the positive set. Considering only gene pairs for which each gene has a phenotypic signature, we obtained a total of 196,122 positives and 2,270,353 negatives. A total of 355,025 gene pairs were not present in either set, and so were not used for evaluation purposes.

### Determination of metric link precisions

For a metric, the highest-scoring *k *gene pairs present in the union of the genes in the positive and negative sets were assigned a precision score:

$$precision(k)=\frac{\left|pos_{k}\right|}{\left|pos_{k}|+|neg_{k}\right|},$$

where |*pos*_*k*_| is the number of gene pairs from the positive evaluation set in the top *k *gene pairs, |*neg*_*k*_| is the number of gene pairs from the negative evaluation set in the top *k *gene pairs, and *k *ranges from 2,500 to 100,000, in increments of 2,500.

### Determination of the significance of the difference between the gene neighborhood functional coherence of the *AGREE *and *PhenoBlast *metrics

Using each gene as a "query gene", we identified the genes with the top 25 most similar phenotypic signatures using each metric. We calculated the precision of each query gene as described above. Query genes with precisions over 0.25 were considered to be "high precision queries."

We defined *D*_*real *_to be the difference between the number of high precision queries using the *AGREE *metric and *The PhenoBlast Metric*. We defined *D*_*rand *_to be the difference between the number of precisions selected randomly with replacement from the distributions of *AGREE *and *PhenoBlast Metric *precisions. The significance of *D*_*real *_was estimated by comparing to the mean (*μ*_*rand*_) and standard deviation (*σ*_*rand*_) computed across 10,000 *D*_*rand*_s, using a standard Z-score transformation:

$$Z=\frac{D_{real}-\mu_{rand}}{\sigma_{rand}}.$$

### Construction of Networks

We constructed a *UPC *network (UNET) and an *AGREE *network (ANET) of equal size by linking gene pairs exceeding a threshold for each metric. We chose a *UPC *threshold of 0.8663, which corresponded to a significance level of *P *< 0.01 for all gene pairs. This significance level was calculated using the Z-score obtained from the mean and standard deviation of the *UPC *metric score across all gene pairs. A total of 32,530 gene pairs exceeded this threshold. We chose a corresponding normalized *AGREE *threshold (0.3531) such that the same number of gene pairs were present in its network.

### Creation of non-redundant functional categories

To compare the functional categories captured by the *AGREE *and *UPC *metrics, we created a set of non-redundant categories. This set was created by first sorting all categories present in the positive evaluation set in order of decreasing size (to capture broader categories). Starting at the top of this list, each category was included in the final non-redundant set only if its gene members did not overlap an already-included category by 50% or more.

### Comparison of functional categories captured by *AGREE *and *UPC*

For each non-redundant functional category *c *in our evaluation set, we calculated the mean pairwise score of all gene pairs contained in that category, *μ*_*c*_, using both the *AGREE *and *UPC *metrics. For a category containing *n*_*c *_genes, we assessed the significance of *μ*_*c *_by comparing to the mean and standard deviation of *μ*_*c *_calculated across 10,000 random gene sets of size *n*_*c *_and performing a standard Z-score transformation.

### Identification of subnetworks

We identified subnetworks in each network by running MODES [24] with settings (d = 0.50, s = 80, c = 0.80, g = 4). Settings were chosen such that all subnetworks contained at least four genes, with a minimum connectivity of 50%.

### Determination of the significance of the difference between the number of enriched phenotypes within ANET and UNET subnetworks

We identified enriched phenotypes within each subnetwork using the hypergeometric distribution (with a cutoff of *P *< 0.01). To assess the significance of the difference in the distributions of the number of enriched phenotypes in the ANET and UNET subnetworks, we compared the difference of their means across all subnetworks. We sampled with replacement 10,000 times from the distributions of enriched phenotype counts for both the ANET and UNET subnetworks, calculating their difference each time. We then calculated the standard deviation of the 10,000 differences. Finally, we performed a Z-score transformation by dividing the true difference in the means by this standard deviation.

### Construction of superimposed networks

We constructed superimposed networks from several large-scale interaction datasets, including a *C. elegans *protein-protein interaction network [46], a eukaryotic protein interaction network that augments the *C. elegans *protein interaction network with orthologous interactions (interologs) mapped from *S. cerevisiae*, *D. melanogaster*, and human protein interactions contained in BioGRID [47], an mRNA coexpression network constructed from *C. elegans*, *S. cerevisiae*, *D. melanogaster*, and human expression data [48,49], and genetic interactions identified from low-throughput experiments that were collected from the literature by WormBase [50]. All interactions assembled from organisms other than *C. elegans *were mapped to predicted worm ortholog pairs using BLASTP [44] with a significance cutoff of *P *< 10^-30^.

### Identification of novel subnetworks

Subnetworks identified in the ANET, UNET, and superimposed network were automatically inspected to determine which types of data significantly link their gene members. For each subnetwork, the significance of the number of links of a specific data type *t *connecting two genes within the subnetwork was calculated using a connectivity significance score. The connectivity significance score for data type *t *for a subnetwork containing *n *genes was calculated as a standard Z-score (*l*_*t *_- *μ*_*t*_)/*σ*_*t*_, where *l*_*t *_is the observed number of links in the subnetwork of type *t*, and *μ*_*t *_and *σ*_*t *_are the mean and standard deviation of the number of links across 1,000 random collections of *n *genes taken from the network constructed from data type *t*. Subnetworks were annotated as enriched for a data source if the connectivity score had an associated *P*-value of 0.01 or less. Novel UNET subnetworks were identified as subnetworks enriched for UNET links, but not enriched for ANET links. Novel *AGREE *multiply-supported subnetworks were identified as subnetworks enriched for ANET links and at least one other link type, while remaining unenriched for UNET links.

### Determination of enriched subnetwork functional annotations

We assigned putative functions to subnetworks by determining their overlaps with categories in our positive validation set. We assessed the significance of the overlap of each subnetwork to a category, *c*, using the *P*-value obtained from the hypergeometric distribution:

$$P\left(X\geq k\right)=\sum_{i=k}^{ns}\frac{\left(\begin{array}{c} n_{c}\\ n_{b} \end{array}\right)\left(\begin{array}{c} n_{s}-n_{c}\\ N-n_{b} \end{array}\right)}{\left(\begin{array}{c} n_{s}\\ N \end{array}\right)},$$

where *n*_*b *_is the number of genes present in both the subnetwork and the category, *n*_*s *_is the size of the subnetwork, *n*_*c *_is the size of the category, and *N *is the number of genes in any category. We estimated hypergeometric *P*-values using the approximation implemented in *R*.

### Calculation of the significance of the deviation of observed phenotype frequency/precision pairs from expected values

To evaluate the intuition that rarer phenotypes might be more informative than frequent phenotypes, we calculated the precision of all pairs of genes displaying each knock-down phenotype using our validation set. We then plotted the precision of each phenotype as a function of its overall background frequency across all genes. To assess the significance of the trend for rarer phenotypes to be more informative, we divided this space into four quadrants based on high/low precision and high/low background frequency. Note that these quadrants correspond to those displayed by the dashed lines in Additional Data File Figure 4b. Any phenotype with a precision exceeding the mean precision across all phenotypes was considered to have "high" precision, and "low" otherwise (likewise for frequencies). We obtained a *P*-value by comparing the observed number of phenotypes in each quadrant to the expected number of phenotypes (assuming a uniform distribution) using a *χ*^2 ^test with three degrees of freedom.

### Evaluation of phenotype correlation dependency

We calculated the correlation between phenotypes as described above in the definition for the *Weighted FDP *metric. We evaluated the intuition that more functional information is conveyed when genes share less-correlated phenotypes than when they share more-correlated phenotypes. To achieve this, we determined the amount of precision gained when genes share an additional uncorrelated phenotype, as opposed to sharing an additional correlated phenotype. We first restricted our analysis to include all gene pairs sharing exactly two phenotypes. For each phenotype *v*, we considered all gene pairs that share exactly that phenotype along with any other second phenotype *w*. We measured the baseline precision *P*_*v*0 _of all gene pairs sharing a phenotype *v *using our evaluation set, as described above. For each phenotype *w*, we then considered gene pairs that share phenotypes *v *and *w*, and calculated their precision *P*_*vw*_. We calculated the significance of the increase in precision obtained by sharing phenotype *w *over the baseline precision using the proportion statistic

$$Z_{vw}=\frac{P_{vw}-P_{v0}}{\sqrt{\frac{P_{v0}(1-P_{v0})}{n_{vw}}}},$$

where *n*_*vw *_is the number of gene pairs sharing phenotypes *v *and *w. Z*_*vw *_can be interpreted as a standard Z-score, and quantifies the degree to which the precision of a gene pair sharing phenotype *v *increases when adding phenotype *w *as opposed to any phenotype in general. For each phenotype pair (*v*, *w*), we plotted *Z*_*vw *_as a function of the correlation of phenotypes *v *and *w*. We restricted analysis to phenotype pairs shared in at least 10 gene pairs.

## Authors' contributions

MW designed and implemented the majority of analyses. CW implemented most metrics. AB analyzed and interpreted novel multiply-supported subnetworks. MW and JS conceived of the study and drafted the manuscript.

## Supplementary Material

Additional file 1 **Phenotype data matrix**. The phenotypic signatures for all 2,376 genes. Click here for file

Additional file 2 **Phenotypes used in this study**. Overview of knock-down phenotypes, including background frequencies, sources, and descriptions. Click here for file

Additional file 3 **Additional metric evaluations**. The first evaluation measures precision as a function of the number of unique genes contained in the network. Additional gene neighborhood evaluations are also included, with varying neighborhood sizes. Results of evaluations using the mean neighborhood precision as a summary statistic instead of the number of high precision neighborhoods are also included. Click here for file

Additional file 4 **Evaluation of phenotype dependencies**. Evaluation of three possible dependencies which may be present in the data, which different metrics could exploit: 1) *number dependency*: sharing a greater number of present phenotypes is positively correlated with sharing a common function; 2) *frequency dependency*: the frequencies of shared phenotypes are inversely related to the probability that two genes share a common function; and 3) *correlation dependency*: genes sharing highly correlated phenotypes are less likely to be functionally related than genes sharing the same number of uncorrelated phenotypes. Click here for file

Additional file 5 **Comparison of enriched functional categories**. List of functional categories enriched for *AGREE *scores only, *UPC *scores only, and for both. Click here for file

Additional file 6 **Superimposed Network**. List of the 89,898 gene interactions contained in the superimposed network, including the data source(s) of each interaction. Click here for file

Additional file 7 **Subnetworks**. Gene subnetworks identified in this study (*AGREE*, *UPC, AGREE *multiply-supported, *UPC *multiply-supported, and molecular machines), along with relevant information pertaining to each subnetwork. Click here for file

Additional file 8 **Comparison of Log Likelihood Scores (LLS) for various data sources**. Plot of the LLS for each *C. elegans *data source contained in the Superimposed Network at varying network sizes. Click here for file
