# Supplementary material for: Information-based methods for predicting gene function from systematic gene knock-downs
Source: BMC Bioinformatics. 2008 Oct 29;9:463. doi: 10.1186/1471-2105-9-463 (PMC2596148; doi:10.1186/1471-2105-9-463)
Supplement: Additional file 2 — Phenotypes used in this study. Overview of knock-down phenotypes, including background frequencies, sources, and descriptions. [file 1471-2105-9-463-S2.doc]

| **Phenotype** | **Abbreviation** | **Kamath03** | **Rual04** | **Simmer03** | **Count** | **Frequency** |
| --- | --- | --- | --- | --- | --- | --- |
| Embryonic lethal | EMB | 785 | 341 | 818 | 1235 | 0.5198 |
| Slow post-embryonic growth | GRO | 574 | 40 | 591 | 1019 | 0.4289 |
| Larval arrest | LVA | 54 | 297 | 679 | 870 | 0.3662 |
| Uncoordinated | UNC | 308 | 30 | 622 | 783 | 0.3295 |
| Sterile | STE | 327 | 282 | 184 | 614 | 0.2584 |
| Protruding vulva | PVL | 111 | 141 | 234 | 424 | 0.1785 |
| Lethal | LET | 154 | 174 | 144 | 403 | 0.1696 |
| Sterile progeny | STP | 84 | 11 | 254 | 325 | 0.1368 |
| Reduced brood size | RBS |  | 316 |  | 316 | 0.133 |
| Body morphological defects | BMD | 151 | 20 | 106 | 241 | 0.1014 |
| Sick | SCK | 142 | 83 | 5 | 222 | 0.0934 |
| Ruptured | RUP | 83 | 18 | 108 | 191 | 0.0804 |
| Dumpy | DPY | 92 | 14 | 121 | 182 | 0.0766 |
| Clear | CLR | 172 | 3 | 2 | 176 | 0.0741 |
| Egg-laying defect | EGL | 68 | 20 | 53 | 133 | 0.056 |
| Thin | THIN | 22 | 35 | 40 | 96 | 0.0404 |
| Small | SMA | 24 | 9 | 57 | 88 | 0.037 |
| Paralyzed | PRL | 47 | 8 | 40 | 87 | 0.0366 |
| Patchy appearance | PCH | 83 |  |  | 83 | 0.0349 |
| Sluggish appearance | SLU | 32 |  | 41 | 71 | 0.0299 |
| Long | LON | 28 | 12 | 23 | 61 | 0.0257 |
| Adult lethal | ADL | 47 |  | 12 | 59 | 0.0248 |
| Molting defect | MLT | 30 | 1 | 10 | 38 | 0.016 |
| Blistering of cuticle | BLI | 7 | 29 | 7 | 38 | 0.016 |
| Pale | PALE |  |  | 22 | 22 | 0.0093 |
| High incidence of males | HIM | 16 | 1 |  | 17 | 0.0072 |
| Oocytes | OOC |  | 17 |  | 17 | 0.0072 |
| Roller | ROL | 6 | 1 | 10 | 15 | 0.0063 |
| Multivulva | MUV | 12 | 2 | 4 | 14 | 0.0059 |
| Kinker | KNK | 1 |  | 2 | 3 | 0.0013 |
| Unique phenotype | UNIQ | 3 |  |  | 3 | 0.0013 |
| Vulvaless | VUL | 3 |  |  | 3 | 0.0013 |
| Hyperactive | HYA | 1 |  | 1 | 2 | 0.0008 |
| Social | SOC |  | 1 |  | 1 | 0.0004 |

**Supplementary Data 2.** **Phenotypes used in the study.**

Each entry indicates the total number of genes with the corresponding knockout phenotype in the given study. The column labeled as ‘Count’ gives the total number of unique genes with the corresponding knockout phenotype. Frequencies are calculated as the fraction of genes (out of 2,376) with the corresponding phenotype.
