# Supplementary material for: Information-based methods for predicting gene function from systematic gene knock-downs
Source: BMC Bioinformatics. 2008 Oct 29;9:463. doi: 10.1186/1471-2105-9-463 (PMC2596148; doi:10.1186/1471-2105-9-463)
Supplement: Additional file 3 — Additional metric evaluations. The first evaluation measures precision as a function of the number of unique genes contained in the network. Additional gene neighborhood evaluations are also included, with varying neighborhood sizes. Results of evaluations using the mean neighborhood precision as a summary statistic instead of the number of high precision neighborhoods are also included. [file 1471-2105-9-463-S3.doc]

**
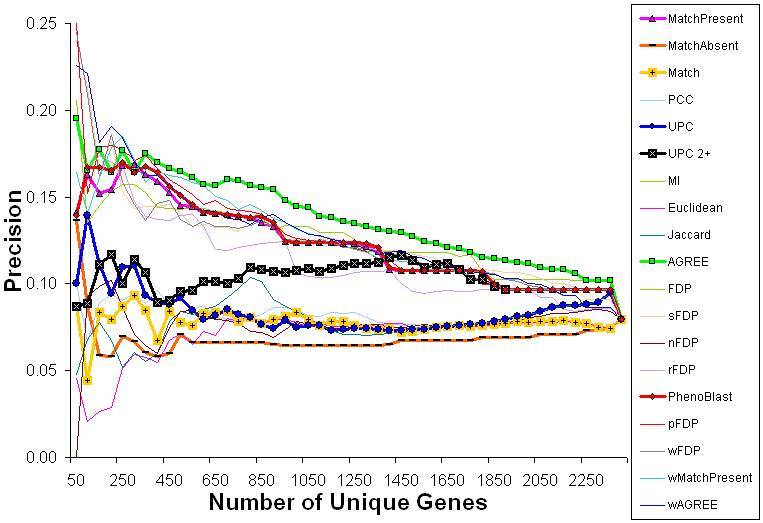
**

**Additional Data File 3a. Precision as a function of number of unique genes**

The precision of the top-scoring gene pairs is shown for each evaluated metric (see Methods.) X-axis indicates the number of unique genes in the network for the given metric. Y-axis indicates the corresponding precision of all gene pairs in the network. Metrics discussed in the text are displayed as bold lines. X-axis range includes all genes with phenotype data present in the validation set.

**
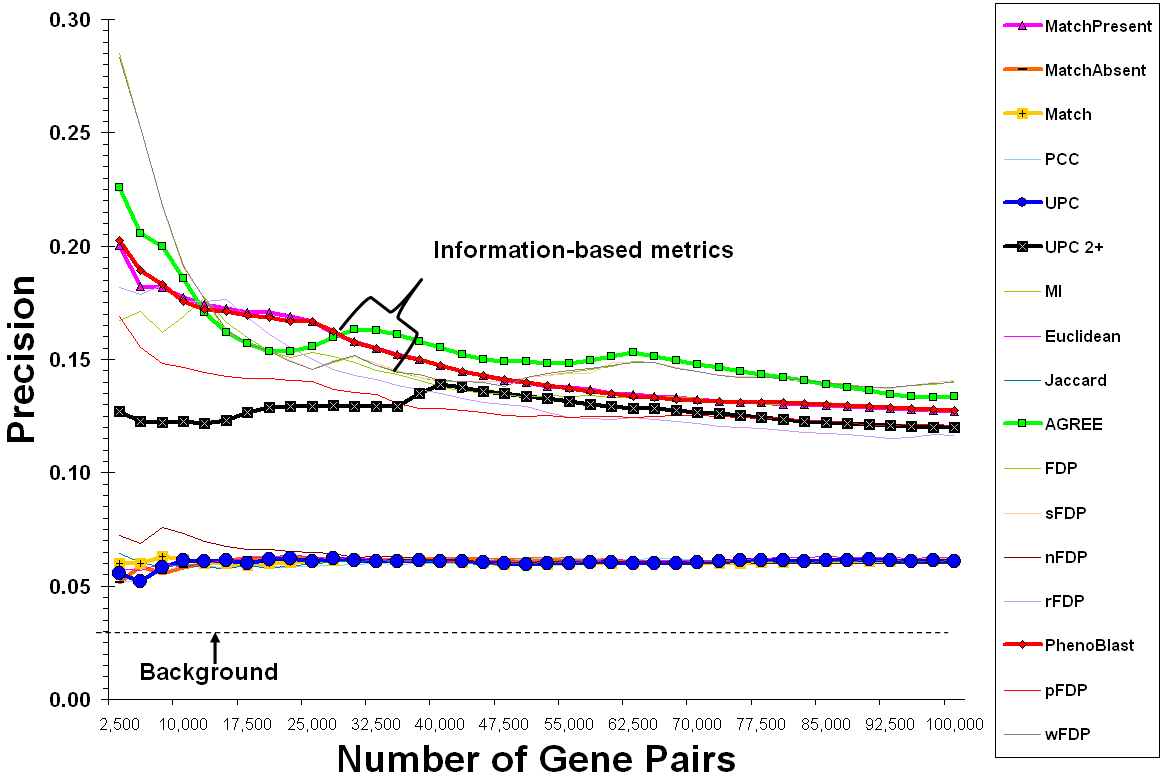
**

**Additional Data File 3b. Yeast gene network precision.** The precision of the top-scoring gene pairs is shown for each evaluated metric (see Methods). Metrics discussed in the text are displayed as bold lines. X-axis indicates the number of top-scoring gene pairs for the given metric. Y-axis indicates the corresponding precision of those gene pairs. Dashed line indicates the background precision of all gene pairs in the dataset. Manually-curated yeast GO categories taken from Huttenhower *et al.* 2006 were used for evaluation.

**Reference:**

Huttenhower C, Hibbs M, Myers C, Troyanskaya OG. A scalable method for integration and functional analysis of multiple microarray datasets. Bioinformatics. 2006 Dec 1;22(23):2890-7. Epub 2006 Sep 27.

|  | **Number of high precision neighborhoods** |
| --- | --- |
| **10** | 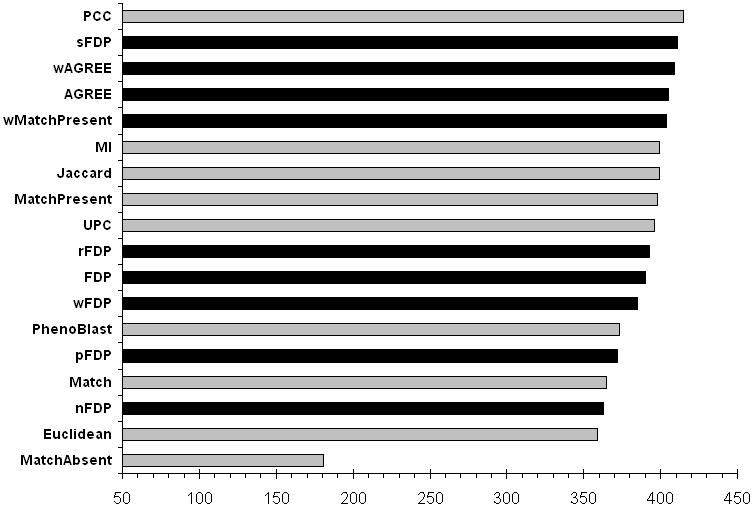 |
| **25** | 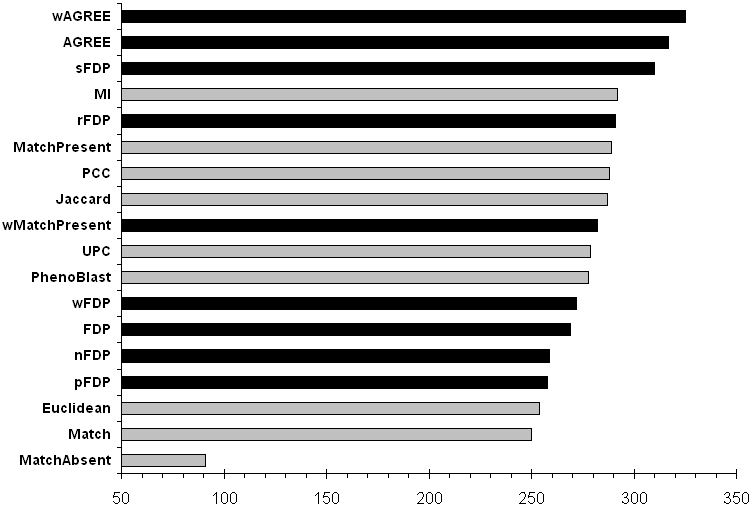 |
| **50** | 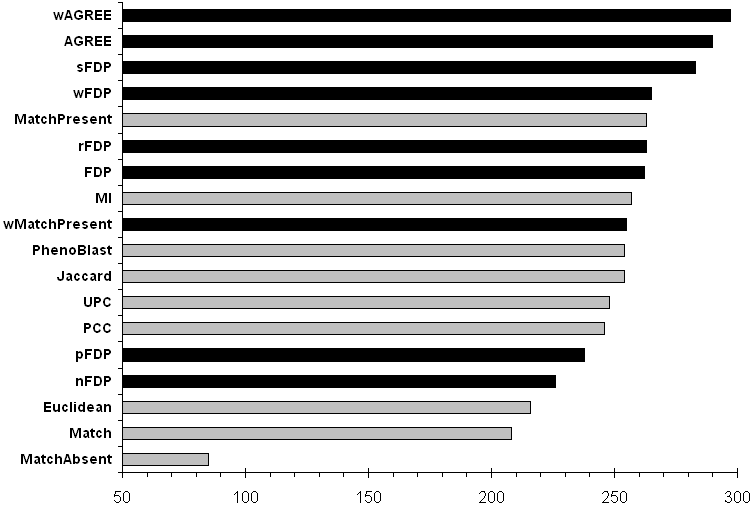 |

|  | **Mean precision of neighborhoods** |
| --- | --- |
| **10** | 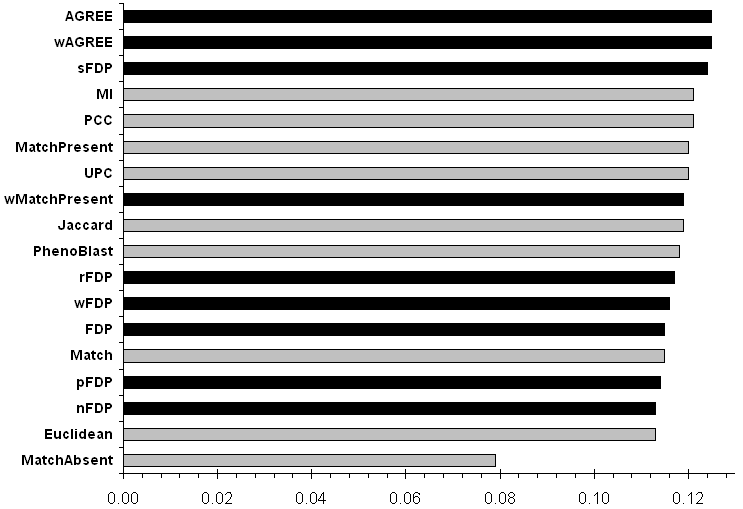 |
| **25** | 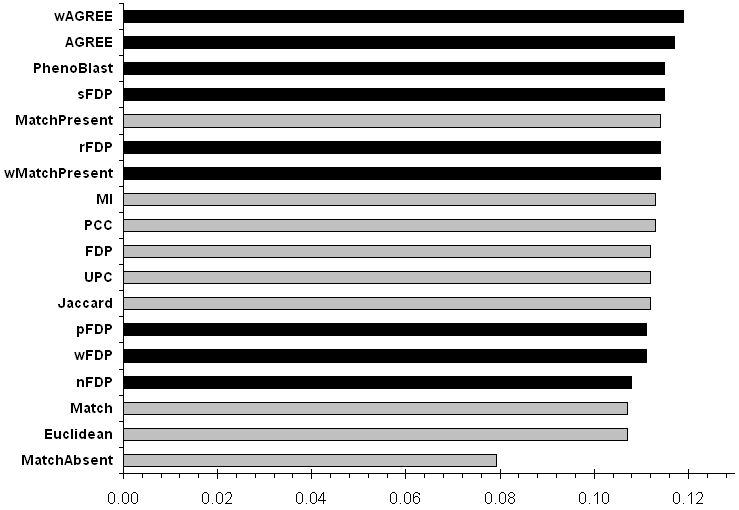 |
| **50** | 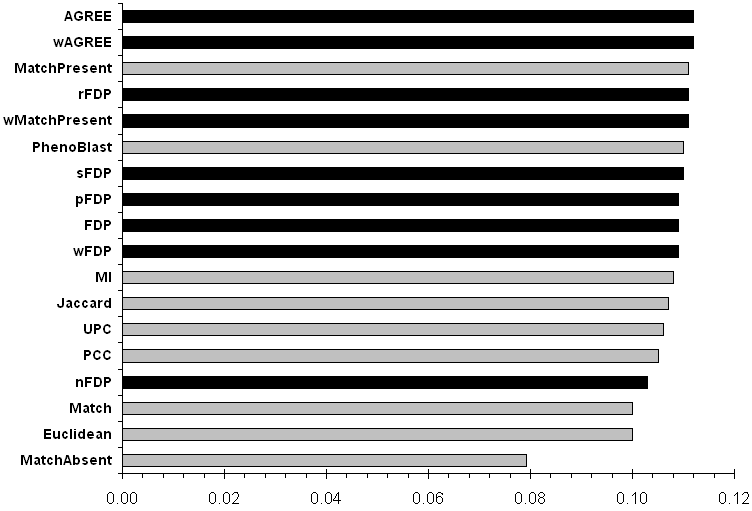 |

**Additional Data File 3c. Additional PhenoBlast Evaluations**

Shown are six evaluations, with varying neighborhood sizes (10, 25, or 50; shown top to bottom), and accuracy measurements (*High Precision* counts the number of neighborhoods with precisions of 0.50 or higher, *Mean Precision* is the mean precision across all neighborhoods.) Information-theoretic metrics are shown as black bars. ‘w’ refers to the weighted version of the corresponding metric.
