# Supplementary material for: Information-based methods for predicting gene function from systematic gene knock-downs
Source: BMC Bioinformatics. 2008 Oct 29;9:463. doi: 10.1186/1471-2105-9-463 (PMC2596148; doi:10.1186/1471-2105-9-463)
Supplement: Additional file 4 — Evaluation of phenotype dependencies. Evaluation of three possible dependencies which may be present in the data, which different metrics could exploit: 1) number dependency: sharing a greater number of present phenotypes is positively correlated with sharing a common function; 2) frequency dependency: the frequencies of shared phenotypes are inversely related to the probability that two genes share a common function; and 3) correlation dependency: genes sharing highly correlated phenotypes are less likely to be functionally related than genes sharing the same number of uncorrelated phenotypes. [file 1471-2105-9-463-S4.doc]

**Phenotypic dependencies predictive of shared gene function**

The differences in performance of the various metrics may reflect the presence of certain dependencies between shared phenotypic observations and shared function. Different metrics may make either explicit or implicit use of such dependencies in their ability to connect functionally related genes. We consider three possible dependencies which may be present in the data that different metrics could exploit: 1) *number dependency*: sharing a greater number of present phenotypes is positively correlated with sharing a common function; 2) *frequency dependency*: the frequencies of shared phenotypes are inversely related to the probability that two genes share a common function; and 3) *correlation dependency*: genes sharing highly correlated phenotypes are less likely to be functionally related than genes sharing the same number of uncorrelated phenotypes.

Number dependency would exist if the frequency of shared function increased with increasing numbers of shared present knock-down phenotypes. All of the metrics, with the exception of *MatchAbsent*, incorporate the number of shared phenotypes into their calculations. We found strong evidence for number dependency in the data; the precision of gene pairs increases monotonically with the number of shared phenotypes (Additional Data File Figure 4a).

Frequency dependency would exist if rare phenotypes convey more functional information than frequently occurring phenotypes. By chance, two arbitrarily chosen genes will share common phenotypes more frequently than rare phenotypes. Accordingly, gene pairs sharing rare phenotypes might be more likely to be co-functional than gene pairs that share common phenotypes. We tested for the existence of frequency dependency by plotting the precision of gene pairs with each knock-down phenotype as a function of the phenotype’s background frequency (Additional Data File Figure 4b). Knock-downs yielding rare phenotypes have a significantly higher proportion of functionally related gene pairs compared to knock-downs resulting in common phenotypes (*P* ~ 0.005, Chi-square test, see Methods). For example, ‘Embryonic lethal’ (Emb) is present in 1,235 (52%) of the genes included in this study. The 761,995 combinations of genes that result in Emb have a precision of only 0.09. This is to be expected, as different essential proteins can be involved in unrelated molecular processes. Conversely, genes sharing the rare ‘Roller’ phenotype (Rol, 0.6% frequency) have a precision of 0.25. Thus, we also find that there is significant evidence for frequency dependency in this data set.

Correlation dependency would exist if uncorrelated phenotypes were more informative indicators of gene function compared to correlated phenotypes. For example, correlation exists between Emb (present in 1,235 genes) and ‘Slow post-embryonic growth’ (Gro, present in 1,019 genes), as they co-occur in the phenotypic profile of 502 genes. As such, in the case that two genes share Emb, little additional information about their common function will be conveyed by knowing that they also share Gro. To test for the presence of this dependency, we calculated the correlation of each pair of phenotypes across all genes (see Methods). We also calculated a statistic measuring the increase in precision achieved by sharing a specific phenotype along with a reference phenotype and compared this to the precision of the reference phenotype with any shared phenotype (see Methods). We found that, for the ranges of phenotype correlations present in the dataset, there is little relation between phenotype correlation and increase in precision (R^2^ value of 0.005, Additional Data File Figure 4c). Most data points fall within the shaded region of Additional Data File Figure 4c, which indicates phenotype pairs with insignificant changes in precision. A total of 71 phenotype pairs have a significant increase in precision, while 73 have a significant decrease. These phenotypes are roughly uniformly distributed across all correlations present in this dataset (Additional Data File Figure 4c). While correlation dependency will almost certainly be present in datasets containing highly correlated phenotypes, these results suggest that moderate levels of correlation between phenotypes (less than 0.4) do not exhibit correlation dependency. Thus, for this dataset, metrics that ignore correlations between phenotypes will not lose much predictive information pertaining to common gene function.

| **A.** | **B.** |
| --- | --- |

**
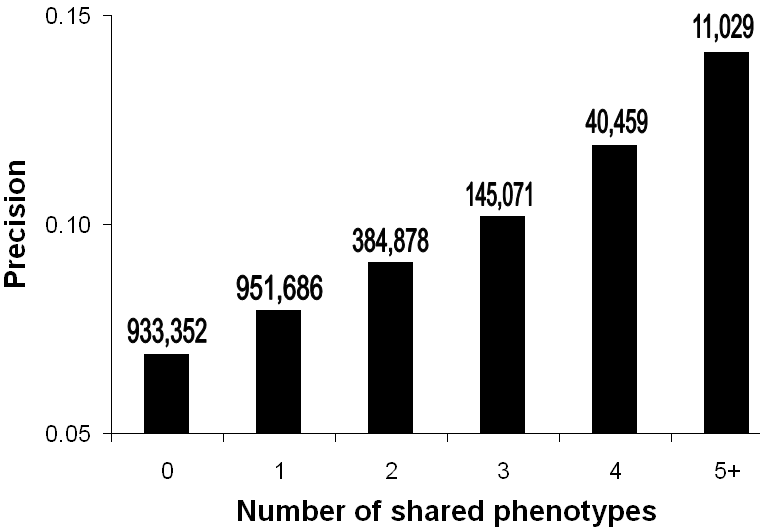
**
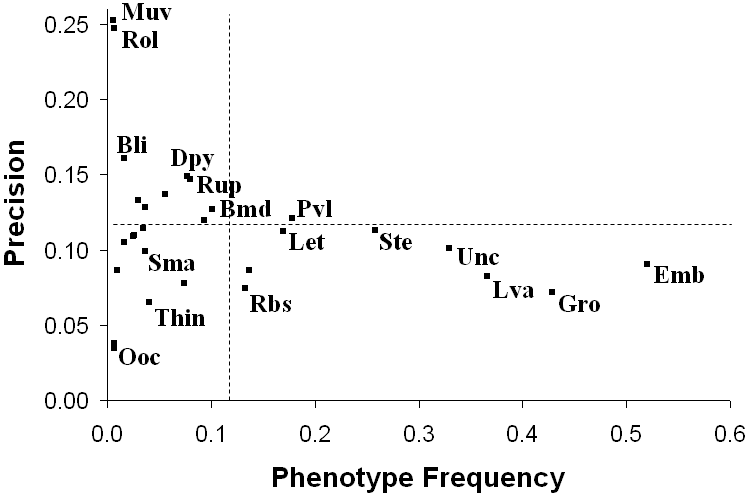


| **C.** |  |
| --- | --- |

**
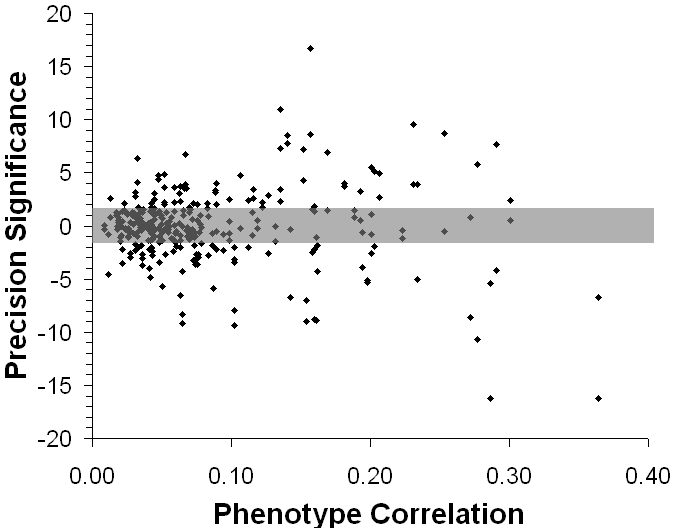
**

**Additional Data File Figure 4. Investigation of phenotypic dependencies. A. Evidence for number dependency: precision increases with the number of shared phenotypes.** All pairs of genes were grouped according to the number of phenotypes that they share (X-axis). The precision of each group is plotted on the Y-axis. Values above bars indicate the number of gene pairs in the group. **B. Evidence for frequency dependency: genes sharing rarer phenotypes have higher precision.** For each phenotype, the precision of all pairs of genes with that phenotype was determined. Precision was measured as in (A). Horizontal dashed line indicates mean precision across all phenotypes. Dashed vertical line indicates mean frequency across phenotypes. **C. Lack of evidence for correlation dependency: precision is unaffected by phenotype correlations.** Shown is the correlation of each pair of phenotypes across all genes (X-axis) plotted against the increase in precision (Y-axis). Shaded region indicates range of Z-scores insignificant at the p = 0.05 level.
