# Supplementary material for: Information-based methods for predicting gene function from systematic gene knock-downs
Source: BMC Bioinformatics. 2008 Oct 29;9:463. doi: 10.1186/1471-2105-9-463 (PMC2596148; doi:10.1186/1471-2105-9-463)
Supplement: Additional file 8 — Comparison of Log Likelihood Scores (LLS) for various data sources. Plot of the LLS for each C. elegans data source contained in the Superimposed Network at varying network sizes. [file 1471-2105-9-463-S8.doc]

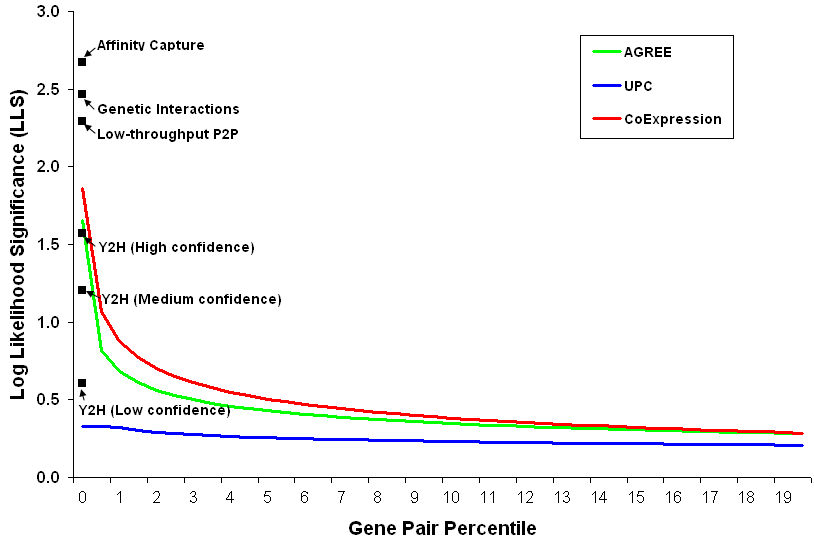


**Additional Data File 8. Comparison of Log Likelihood Scores (LLS) for various data sources.** Using the methods of Lee *et al.* 2008, we calculated the Log Likelihood Score (LLS) for each *C. elegans* data source used in our Superimposed Network. Shown is the LLS for each data source, as a function of the gene pair percentile (i.e., the height of a line at an X-axis value of 1 indicates the LLS of the top 1% of gene pairs for that data source.) Data sources such as protein-protein and genetic interactions are binary, and so are displayed as points instead of lines. Y2H points refer to yeast two-hybrid interactions taken from Li *et al.* 2008. Confidence scores correspond to the confidence levels reported by the authors: ‘High confidence’ corresponds to ‘Core-1’ interactions, ‘Medium’ corresponds to ‘Core-2’, and ‘Low’ corresponds to ‘Non-Core’.
